# Dysferlin and Other Non-Red Cell Proteins Accumulate in the Red Cell Membrane of Diamond-Blackfan Anemia Patients

**DOI:** 10.1371/journal.pone.0085504

**Published:** 2014-01-14

**Authors:** Esther N. Pesciotta, Sira Sriswasdi, Hsin-Yao Tang, David W. Speicher, Philip J. Mason, Monica Bessler

**Affiliations:** 1 Department of Pediatrics, Children’s Hospital of Philadelphia, Philadelphia, Pennsylvania, United States of America; 2 The Center for Systems and Computational Biology and Molecular and Cellular Oncogenesis Program, The Wistar Institute, Philadelphia, Pennsylvania, United States of America; 3 Genomics and Computational Biology Graduate Group, University of Pennsylvania, Philadelphia, Pennsylvania, United States of America; 4 Department of Internal Medicine, University of Pennsylvania, Philadelphia, Pennsylvania, United States of America; University of Louisville, United States of America

## Abstract

Diamond Blackfan Anemia (DBA) is a congenital anemia usually caused by diverse mutations in ribosomal proteins. Although the genetics of DBA are well characterized, the mechanisms that lead to macrocytic anemia remain unclear. We systematically analyzed the proteomes of red blood cell membranes from multiple DBA patients to determine whether abnormalities in protein translation or erythropoiesis contribute to the observed macrocytosis or alterations in the mature red blood cell membrane. In depth proteome analysis of red cell membranes enabled highly reproducible identification and quantitative comparisons of 1100 or more proteins. These comparisons revealed clear differences between red cell membrane proteomes in DBA patients and healthy controls that were consistent across DBA patients with different ribosomal gene mutations. Proteins exhibiting changes in abundance included those known to be increased in DBA such as fetal hemoglobin and a number of proteins not normally found in mature red cell membranes, including proteins involved in the major histocompatibility complex class I pathway. Most striking was the presence of dysferlin in the red blood cell membranes of DBA patients but absent in healthy controls. Immunoblot validation using red cell membranes isolated from additional DBA patients and healthy controls confirmed a distinct membrane protein signature specific to patients with DBA.

## Introduction

Diamond Blackfan Anemia (DBA) is a rare, congenital anemia characterized by a paucity of erythroid progenitor cells in the bone marrow, reticulocytopenia, and an increased size (macrocytosis) of the remaining circulating red blood cells (RBCs), with elevated adenosine deaminase activity and increased fetal hemoglobin. [1] In the majority of patients, DBA is caused by a heterozygous mutation in a gene that encodes one of several ribosomal proteins in either the small or large ribosomal subunits. To date, mutations in *RPS19, RPS17, RPS24, RPL35A, RPL5, RPL11, RPS7, RPS10, RPS26, RPL15* and *RPL26* have been associated with DBA, with *RPS19* being most frequently mutated, accounting for 20–25% of known mutations. [2] Currently, mutations in these ribosomal genes account for disease in at least 50–70% of patients with DBA. [2], [3] The phenotypic presentation of DBA is highly variable, ranging from 1) asymptomatic, to 2) a macrocytic anemia that improves with corticosteroid treatment, to 3) patients whose anemia is so severe that they are dependent on RBC transfusions. Interestingly, the degree of anemia often varies during the course of disease and is usually not present at birth but develops during the first year of life. Anemia might be initially responsive to corticosteroids but may become so severe that the patient becomes transfusion-dependent later in life. In about 15% of patients the anemia improves spontaneously to the extent that no further treatment is required [4].

While the genetic basis of DBA is well characterized, the mechanism by which impaired ribosome biosynthesis leads to macrocytic RBCs with elevated levels of fetal hemoglobin and adenosine deaminase activity are still unclear. DBA mainly affects the erythroid lineage and does not respond to treatment with erythropoietin. [5] In vitro colony forming assays with bone marrow cells from patients with DBA demonstrated a decreased proliferation and increased apoptosis in erythroid colonies that pinpoints the defect to the erythropoietin-dependent stages of RBC differentiation. [6], [7], [8] Additionally, in vitro culture studies and studies in animal models of DBA demonstrated that inactivation of p53 improves or even restores RBC production, suggesting a p53 dependent cell cycle checkpoint may be involved in pathogenesis [9], [10].

Previous studies have investigated gene expression in purified bone marrow populations isolated from DBA patients in remission, [11] a mouse model using fetal liver erythroid cells with knockdown of RPS19, [12] and a transgenic mouse model with an RPS19 mutation that causes a DBA-like phenotype. [13] Results from these studies highlighted the upregulation of apoptotic genes such as *TNFRSF10B* and *FAS* [11] as well as decreased expression of transcription factor *MYB* [11], [12] and its target, *c-kit*. [12], [13] Because DBA is a disease of defective ribosome biogenesis, we hypothesized that some of the RBC abnormalities may be due to faulty translation or protein sorting and degradation during erythroid maturation. Hence, quantitative label-free proteomic analysis of RBC membranes from DBA patients could provide new insights into downstream effects of altered ribosome biogenesis in RBC progenitors that would not be apparent in gene expression analysis. Additionally, any differences in the RBC proteome could lead to new diagnostic or prognostic DBA biomarkers or identify novel therapeutic targets.

In this study, we performed label-free proteomic analysis of RBC membranes from four DBA patients with differing ribosomal gene mutations and compared the results with those from matched healthy controls. A number of proteins showed altered abundance levels in DBA RBC membranes compared with controls, however, none of the major RBC membrane proteins exhibited significant abundance changes. The most dramatic difference was the presence, in DBA RBCs, of proteins not normally observed in RBC membranes, such as dysferlin and members of the MHC class I pathway. Furthermore, most observed protein changes did not appear to correlate with specific ribosomal protein gene mutations. Our results suggest that DBA RBCs have a characteristic pattern of RBC membrane protein composition that differs markedly from the proteome of normal RBC membranes and points towards the activation of a cellular stress response that appears to be relatively specific to DBA.

## Materials and Methods

### Ethics Statement

Whole blood was collected from individuals after informed written consent was obtained. All experiments were in accordance with the principles expressed in the Declaration of Helsinki with procedures approved by the IRB of the Children’s Hospital of Philadelphia and University of Pennsylvania.

### RBC Membrane Processing

Approximately 5–10 mL whole blood was collected in K_2_EDTA tubes and all materials were kept at 0–4°C throughout processing to minimize proteolysis. RBC purification and membrane preparation was performed as previously described. [14] Briefly, RBCs were purified by filtering with a leukocyte depletion filter (Plasmodipur®, Accurate Chemical & Scientific Corp) and washing the cells 4 times in PBS with subsequent removal of the top layer of RBC. RBC membranes were prepared by lysing with hypotonic buffer (5 mM sodium phosphate, 1 mM EDTA, 0.1 mM DFP (diisopropylfluorophosphate), pH 8.0) followed by 4–5 washes in the same buffer, until white RBC membranes were obtained. Protein concentration was determined using the Modified Lowry Protein Assay (Thermo Scientific). Aliquots of each sample were stored immediately at −80°C until future use. Pools of control RBC membranes were prepared by combining equal protein amounts of each individual control sample.

### Sample Preparation and LC-MS/MS

RBC membrane fractionation was performed by separating 12 µg total protein on 10% Bis-Tris mini gels with MOPS SDS buffer (Invitrogen) until the dye front reached 3 cm. Three parallel gel lanes of the same sample were sliced into 30×1 mm fragments and corresponding slices were pooled. Pooled gel slices were reduced, alkylated, digested with trypsin and analyzed by LC-MS/MS as previously described [14].

### Data Processing and Label-Free Analysis

Data was processed through the Rosetta Elucidator software package (Ver. 3.3, Rosetta Biosoftware). Using this software, the raw data was aligned across LC-MS/MS full scans for corresponding gel slices to align identical MS features (discrete m/z signals at a given retention time), which were quantitated based on peak heights of ion intensities, as previously described. [15], [16], [17] Corresponding peptide sequences were identified using the SEQUEST algorithm (Ver. 28, rev. 13, University of Washington) and the human UniRef 100 database (Ver. June, 2011) that was expanded to include expected environmental contaminants. [17] Proteins were annotated using ProteinTeller scores >0.95 and PeptideTeller scores >0.8 to obtain a protein false discovery rate of <0.5%. Raw data and search results can be found in the Peptide Atlas data repository with the dataset identifier PASS00334 (www.peptideatlas.org).

### Statistical Analysis

Protein lists were generated with a 2 peptide minimum and common protein contaminants (e.g. trypsin, keratins, etc.) were excluded. An intensity cutoff of 1×10^4^ was applied to peptides to eliminate erroneous protein intensities attributed to background noise. Final protein intensities were calculated by summing the associated peptide intensities followed by normalizing based on the median log10 intensity ratio of DBA/Control. In samples where no protein was detected, to avoid division by zero a fold change threshold of ±100 was applied. 95% confidence intervals were calculated by fitting the standard deviation versus the mean protein intensities of both control datasets to an exponential decay function. [14] This confidence interval was then transposed onto a plot of protein intensities from pair wise DBA versus control to identify significantly changed proteins.

### Western Blot Validation

Select proteins were chosen to validate proteomics label-free quantitation and assess relative protein levels in additional DBA patient samples not analyzed by LC-MS/MS. Anti- HLA Class I ABC (ab70328), SPTLC1 (ab176706), SPTLC2 (ab23696), β-actin (ab8227), ICAM4 (ab67943), and CR1 (EPR6601) antibodies were purchased from Abcam. The anti-Dysferlin antibody, NCL-Hamlet, was purchased from NovoCastra, anti-ADD3 (17585-1-AP) was purchased from Proteintech Group, and anti-MVP (2H3-1A6) was purchased from Novus Biologicals. Goat anti-mouse and goat anti-rabbit secondary antibodies conjugated with HRP were purchased from Abcam (ab6789) and Pierce (31460), respectively. Blots were developed using Amersham ECL Prime (GE Healthcare).

### Confocal Microscopy

The procedure for RBC fixing and permeabilization was adapted from a study by Franco et al and performed at room temperature. [18] Washed RBCs were fixed in 1 mL of freshly prepared 0.5% acrolein (VWR) in PBS for 5 min followed by quenching with 4 washes of cold 0.1 M glycine/PBS. Cells were permeabilized with equal volume of 0.1% Triton-X 100/PBS for 5 minutes followed by 3 washes with blocking buffer (0.1 M glycine/PBS with 0.2% cold water fish gelatin (Sigma-Aldrich)) and incubated in the blocking buffer for a minimum of 1 hour. RBCs were incubated with a mouse anti-dysferlin antibody and rabbit anti-α-spectrin antibody, [19] for a minimum of 40 minutes and subsequently washed 3 times with blocking buffer. RBCs were then incubated for 40 minutes with a goat anti-mouse antibody conjugated with Alexa Fluor® 488 and a goat anti-rabbit antibody conjugated with Alexa Fluor® 633 (Molecular Probes) followed by 3 washes. Cells were mounted onto poly-l-lysine coated slides with Aqua-Mount (Thermo-Scientific). Images were obtained with a Leica TCS SP5 II scanning laser confocal microscope (Leica Microsystems) equipped with AOBS and HyD detectors.

## Results

### Evaluation of DBA Patient Proteomes

**Figure 1:**
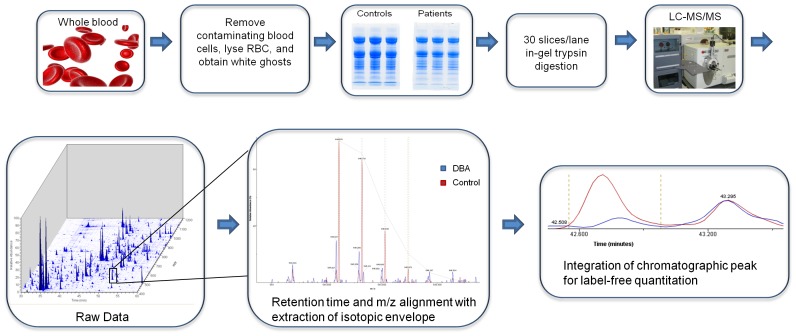
Schematic of sample processing and subsequent proteomic and data analysis pipeline.

A total of four transfusion-independent DBA patients were chosen for proteomic analysis (Table 1). We focused on the analysis of RBC membranes, i.e. “white ghosts”, comprising mainly membrane and membrane skeleton proteins. RBC membrane preparations from two female DBA patients (D1 and D2) were compared to two RBC membrane samples (C1 and C2) each pooled from three age, sex and ethnically matched healthy control donors (C1a-C1c and C2a-C2c). Similarly, two male DBA patients (D3 and D4) were compared to two pools (C3 and C4) of two matched healthy control donors (C3a-C3b and C4a-C4b). Control pools were used to average natural variations in protein levels while at the same time keeping the scope of the proteomics analyses within practical limits. For each DBA patient, two blood samples drawn at least three months apart were prepared separately for replicate analyses, with the exception of D2 in which two aliquots of the same blood draw were processed in parallel because only one blood sample was available. Pair wise comparisons were performed using normalized average protein intensities from DBA patient replicates and appropriate matched control pools. An overview of the experimental scheme is shown in Figure 1. The comparison between D1 and D2 vs. controls C1 and C2 resulted in 1304 high confidence protein identifications, and the comparison between D3 and D4 vs. C3 and C4 identified 1116 proteins. Significant changes in protein abundance were determined by calculating 95% confidence intervals from the control pools, which were imposed onto protein intensity plots for each DBA patient and their appropriate control pools (Figures S1 and S2 in File S1). Significantly changed proteins were identified with log10 intensities outside of the 95% confidence intervals that were defined using the healthy control datasets. Fold changes for those significantly changed proteins were then calculated from the ratio of log10 protein intensities of DBA patients versus matched controls.

**Table 1:** DBA Patient Population.

	Age	Sex	Ethnicity	Gene Mutation	Treatment	Hgb* (g/dL)	RBC* (MIL/µL)	MCV* (fL)
D1	32	F	Caucasian	RPS19, nonsense	None†	7.4	1.8	115
D2	22	F	Caucasian	RPL5, splice site	None†	10.2	3.1	93
D3	17	M	Caucasian	Unknown	Prednisone	8.4	2.2	120
D4	16	M	Caucasian	RPS19, splice site	Prednisone	13.5	4.0	98
D5	5	F	African American	Unknown	Prednisone	10.8	4.0	84
D6	22	F	Caucasian	RPS17, frame shift	Prednisone	14.6	4.2	103
D7	14	M	Caucasian	RPL11, frame shift	Prednisone	11.5	3.7	98
D8	1	F	Caucasian	RPL5, frame shift	None	9.7	3.5	90
D9	3	F	Caucasian	Unknown	Prednisone	13.8	4.2	98
C1a	24	F	Caucasian					
C1b	28	F	Caucasian	Pool C1 for comparison to D1, D2				
C1c	28	F	Caucasian					
C2a	33	F	Caucasian					
C2b	35	F	Caucasian	Pool C2 for comparison to D1, D2				
C2c	38	F	Caucasian					
C3a	22	M	Caucasian	Pool C3 for comparison to D3, D4				
C3b	22	M	Caucasian					
C4a	20	M	Caucasian	Pool C4 for comparison to D3, D4				
C4b	21	M	Caucasian					

Normal ranges for Hgb: 13.5–17.5 males, 12.0–15.5 females; RBC 4.3–5.7 males, 3.9–5.0 females; MCV 80–100.

Patients D1 and D2 were transfused in the past.

### Comparative Analysis of Significantly Changed Proteins

To further improve the reliability of protein abundance changes, we focused on proteins identified and quantitated by at least three peptides and that were found to be significantly changed in two or more DBA patients based on the 95% confidence intervals described above. Using these criteria, a total of 30 proteins were increased in the RBC membranes of DBA patients (Table 2). Six proteins, dysferlin (DYSF), transporter 1 ATP binding cassette sub-family B (TAP1), major vault protein (MVP), major histocompatibility complex class I A (HLAA) and serine palmitoyltransferase 1 and 2 (SPTLC1, SPTLC2) were found to be present in all four DBA patients but were not detected in the control pools. Additional members of the MHC class I pathway such as β-2-microglobulin (B2M), antigen peptide transporter 2 (TAP2), MHC class I antigen (HLAB), and tapasin were also significantly elevated in DBA patients. C-1-tetrahydrofolate synthase (MTHFD1), gamma-adducin (ADD3), and epoxide hydrolase (EPHX1) were also significantly elevated in three of the four DBA patients tested. Fetal hemoglobin (HbG1 and HbG2), a known marker of DBA RBCs, was elevated in three of the four DBA patients tested. Interestingly, prior DBA studies focused on total hemoglobin, while the hemoglobin fraction analyzed here is that tightly associated with the membrane. [20] Prior studies suggest that the membrane-associated fraction of hemoglobin reflects the composition of the soluble fraction, although this remains to be verified [21], [22].

**Table 2:** Proteins Significantly Increased in Two or More DBA Patients.

UniProt Accession Number	GeneName	Protein Description	PeptideCount	Fold ChangeD1	Fold ChangeD2	Fold ChangeD3	Fold ChangeD4	Outside 95% Interval
O75923	DYSF	Dysferlin	96	[100]*	[100]	[100]	[100]	D1–4
B2R9F3	TAP1	Transporter 1 ATP-binding cassette sub-family B	4	[100]	[100]	[100]	[100]	D1–4
Q14764	MVP	Major vault protein	10	[100]	[100]	[100]	[100]	D1–4
P01891	HLAA	Major histocompatibility complex, class I, A	11	[100]	[100]	[100]	[100]	D1–4
O15269	SPTLC1	Serine palmitoyltransferase 1	5	[100]	[100]	[100]	[100]	D1–4
O15270	SPTLC2	Serine palmitoyltransferase 2	9	[100]	[100]	[100]	[100]	D1–4
P61769	B2M	Beta-2-microglobulin	3	14	9.4	6.2	3.1	D1, 2, 3
D3GKD9	HBG2	G-gamma globin Paulinia variant	12	5.3	1.2	40	8.5	D1, 3, 4
D9YZU8	HBG1	Hemoglobin, gamma	4	8.7	−2.9	34	7.0	D1, 3, 4
P07099	EPHX1	Epoxide hydrolase 1	24	1.7	3.0	4.9	3.9	D2, 3, 4
P11586	MTHFD1	C-1-tetrahydrofolate synthase, cytoplasmic	52	2.1	3.9	3.4	4.9	D2, 3, 4
Q9UEY8	ADD3	Gamma-adducin	21	2.8	6.4	4.3	2.3	D1, 2, 3
P12829	MYL4	Myosin light polypeptide 4	19	2.9	−5.7	5.1	1.0	D1, 2, 3
Q9Y3A5	SBDS	Shwachman-Bodian-Diamond syndrome protein	10	3.6	8.1	4.7	2.5	D2, 3
Q9UNQ0	ABCG2	ATP-binding cassette sub-family G member 2	19	2.7	2.9	1.8	1.2	D1, 2
Q59H06	TAP2	Antigen peptide transporter 2	6	–	–	[100]	[100]	D3, 4
Q53GD8	FKBP3	Peptidyl-prolyl cis-trans isomerase	10	1.4	3.4	5.9	2.9	D2, 3
Q38PK6	HLAB	MHC class I antigen	7	[100]	[100]	3.2	2.1	D1, 2
Q4LE45	MYH10	Myosin, heavy chain 10, non-muscle	71	3.8	1.1	9.2	1.4	D1, 3
P48556	PSMD8	26S proteasome non-ATPase regulatory subunit 8	17	2.7	−2.0	42	4.4	D1, 3
P28062	PSMB8	Proteasome subunit beta type-8	9	12	6.1	2.3	−1.4	D1, 2
P28065	PSMB9	Proteasome subunit beta type-9	4	[100]	[100]	–	–	D1, 2
P22694	PRKACB	cAMP-dependent protein kinase catalytic subunit beta	8	4.2	8.1	5.7	2.1	D1, 2
P15311	EZR	Ezrin	28	1.1	3.2	5.9	2.7	D2, 3
O75935	DCTN3	Isoform 3 of Dynactin subunit 3	8	4.7	4.5	1.5	−3.8	D1, 2
O15533	TAPBP	Tapasin	4	[100]	[100]	–	–	D1, 2
E9PPD9	EPB41L2	Band 4.1-like protein 2	43	2.9	2.7	1.1	−1.1	D1, 2
E7EX90	DCTN1	Dynactin subunit 1	64	3.2	3.1	2.6	−1.3	D1, 2
P31321	PRKAR1B	cAMP-dependent protein kinase type I-beta regulatory subunit	10	1.3	6.8	[100]	[100]	D2, 3
O75069	TMCC2	Transmembrane and coiled-coil domain family 2	22	4.7	1.6	4.4	1.3	D1, 3

For bracketed numbers, a maximum fold change threshold of 100 was used for proteins not detected in controls.

Table 3 lists the 32 proteins that were significantly decreased in two or more DBA patients. Consistent with the above fHb results, the adult forms of membrane-bound hemoglobin (HbB, HbD, and HbA) were decreased in all four DBA patients compared to controls. Complement receptor 1 (CR1) was not detected in any of the DBA patients but was present in all of the control pools. Several other proteins were decreased in three of the four DBA patients including phosphoribosyl pyrophosphate synthase-associated protein 2 (PRPSAP2), class IVb beta tubulin (TUBB2C), tubulin alpha-ubiquitous chain (TUBA1B), and intercellular adhesion molecule 4 (ICAM4).

**Table 3:** Proteins Significantly Decreased in Two or More DBA Patients.

UniProt Accession Number	GeneName	Protein Description	PeptideCount	FoldChange D1	FoldChange D2	FoldChange D3	FoldChange D4	Outside 95% Interval
Q8IUL9	HBB	Hemoglobin beta chain	13	−2.6	−2.2	−2.7	−3.8	D1–4
P02042	HBD	Hemoglobin subunit delta	8	−3.1	−3.1	−3.4	−4.5	D1–4
E9PDY4	CR1	Complement receptor type 1	11	[−100]*	[−100]	[−100]	[−100]	D1–4
O60256	PRPSAP2	Phosphoribosyl pyrophosphate synthase-associated protein 2	17	1.3	−4.3	−19	[−100]	D2, 3, 4
Q8IWP6	TUBB2C	Class IVb beta tubulin	11	−1.0	−7.9	−12	−35	D2, 3, 4
P68363	TUBA1B	Tubulin alpha-1B chain	10	−1.2	−5.9	−8.4	−30	D2, 3, 4
Q14773	ICAM4	Intercellular adhesion molecule 4	9	−9.3	−3.9	−3.0	−1.5	D1, 2, 3
P69905	HBA1	Hemoglobin subunit alpha	18	−2.7	−2.3	−1.8	−2.7	D1, 2, 4
P07900	HSP90A	Heat shock protein HSP 90-alpha	35	−1.2	−3.8	1.4	−7.0	D2, 4
E7ESC6	XPO7	Exportin-7	33	−1.8	−5.5	1.9	−5.0	D2, 4
A8K690	STIP1	Stress-induced-phosphoprotein 1	30	−1.5	−6.7	2.1	−7.2	D2, 4
P04040	CAT	Catalase	30	−1.9	−7.8	1.7	−4.9	D2, 4
P61201	COPS2	COP9 signalosome complex subunit 2	23	1.1	−3.4	1.1	−7.9	D2, 4
P22314	UBA1	Ubiquitin-activating enzyme E1	22	−1.0	−4.1	1.4	−7.1	D2, 4
P00352	ALDH1A1	Retinal dehydrogenase 1	20	−3.3	1.6	−1.4	−8.1	D1, 4
P32119	PRDX2	Peroxiredoxin-2	17	−1.7	−5.1	1.1	−4.9	D2, 4
P50502	ST13	Hsc70-interacting protein	14	−1.7	−5.3	1.5	−6.7	D2, 4
Q06830	PRDX1	Peroxiredoxin-1	14	−1.4	−5.3	−1.2	−8.3	D2, 4
Q5TDH0-3	DDI2	Isoform 3 of DNA-damage inducible 1 homolog 2	14	−1.8	−5.2	−1.5	−15	D2, 4
Q92905	COPS5	COP9 signalosome complex subunit 5	12	1.2	−3.8	−2.0	−13	D2, 4
P17858	PFKL	6-phosphofructokinase, liver type	11	−1.2	−5.3	−5.0	−13	D2, 4
P45974	USP5	Ubiquitin carboxyl-terminal hydrolase 5	11	−1.2	−4.5	1.6	−6.8	D2, 4
Q15631	TSN	Translin	11	−1.2	−4.5	−1.8	−15	D2, 4
Q5T9B7	AK1	Adenylate kinase 1	11	−2.7	−1.6	−8.0	−14	D3, 4
Q12907	LMAN2	Vesicular integral-membrane protein VIP36	10	1.2	1.4	−9.4	−4.2	D3, 4
P04792	HSPB1	Heat shock protein beta-1	7	[−100]	−4.3	−6.6	[−100]	D1, 4
Q9H400	LIME1	Lck-interacting transmembrane adapter 1	7	−15	−9.4	–	–	D1, 2
Q8IUI8	CRLF3	Cytokine receptor-like factor 3	6	−1.6	−8.1	1.5	−7.6	D2, 4
P48507	GCLM	Glutamate-cysteine ligase regulatory subunit	5	−2.3	1.5	−6.5	−19	D3, 4
Q96IU4	ABHD14B	AB hydrolase domain-containing protein 14B	5	–	–	−41	−26	D3, 4
Q13113	PDZK1IP1	PDZK1-interacting protein 1	3	−9.8	−12	−5.4	−3.4	D1, 2
Q9Y4P8	WIPI2	WD repeat domain phosphoinositide-interacting protein 2	3	−	−	−16	−15	D3, 4

For bracketed numbers, a minimum fold change threshold of −100 was used for proteins not detected in patients.

**Figure 2:**
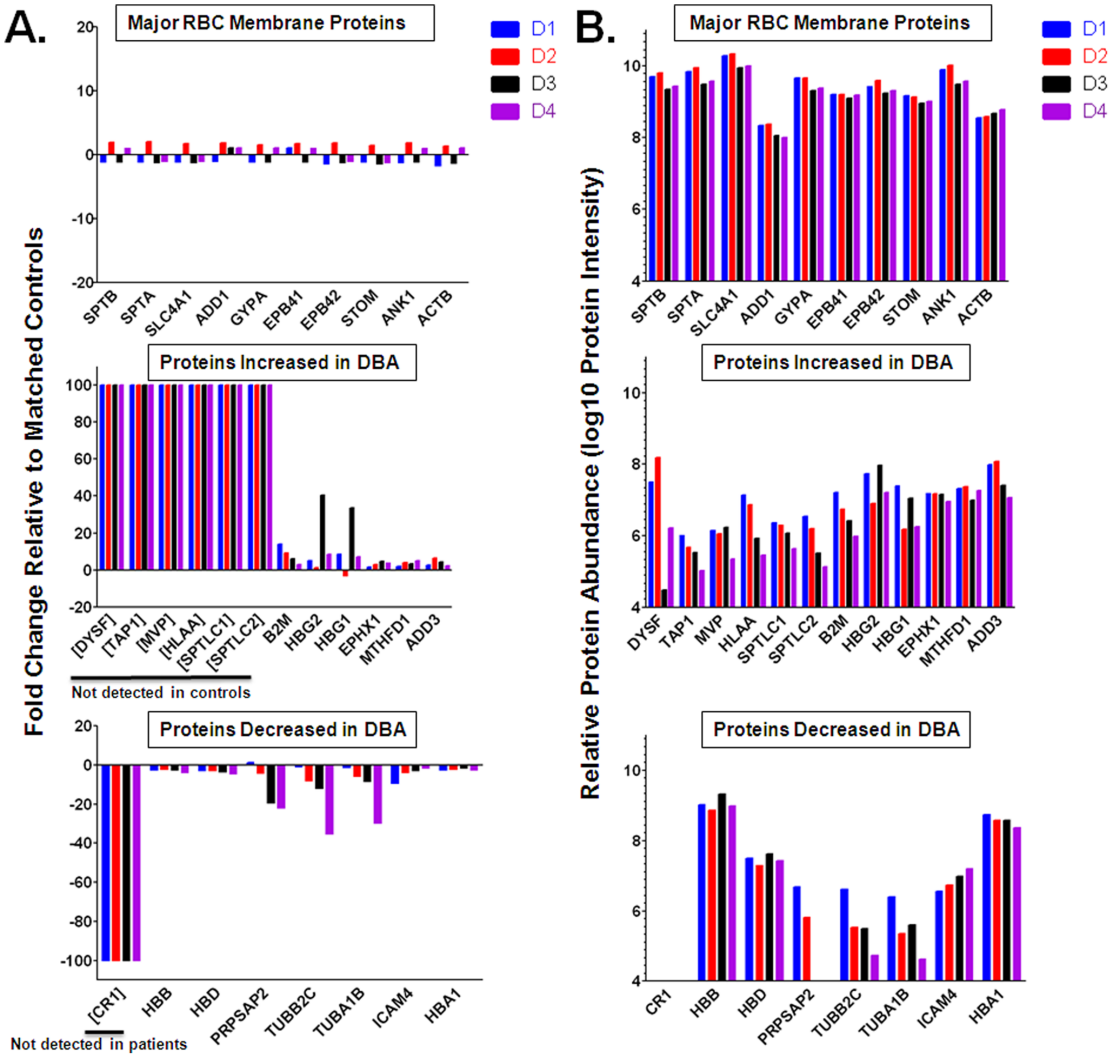
Fold change and relative abundance of significantly changed proteins with comparison to major RBC membrane proteins. A) Fold change relative to controls shows minimal variability for normal, major RBC membrane proteins and more variability between patients for the proteins that were significantly changed in three or more DBA patients. Bracketed proteins were not detected and apparent fold change is based on a fold change threshold of ±100. B) Magnitude of protein abundance in RBCs were calculated for each protein based on the sum of the top three peptide intensities. Significantly changed proteins ranged from low to high abundance as compared to major RBC membrane proteins that were present in high abundance.

Interestingly, major RBC membrane proteins including α-spectrin (SPTA), β-spectrin (SPTB), band 3 (SLC4A1), α-adducin (ADD1), glycophorin A (GYPA), band 4.1 (EPB41), band 4.2 (EPB42), stomatin (STOM), ankyrin (ANK1), and β-actin (ACTB) did not show significant changes in abundance in DBA patients (Tables 2 and 3, Figure 2). The minimal fold changes in these normal major membrane proteins provide an independent indication that experimental variation in protein quantitation is low. In contrast, proteins that were significantly changed in three or more DBA patients in Tables 2 and 3 were highly variable across DBA patients (Figure 2A).

The above comparisons evaluated how individual proteins changed relative to that same protein in the controls. To assess abundance levels across proteins, relative abundance was estimated for each protein by summing the top three peptide intensities to minimize bias due to protein size and variations in peptide signal intensities in the mass spectrometer. [23] Figure 2B shows the relative protein intensities for DBA patients of major RBC proteins as well as a subset of proteins from Tables 2 and 3 that were significantly changed in three or more DBA patients. The proteins differentially expressed in DBA patients were of intermediate intensities (10^4^–10^8^) compared to the most abundant RBC membrane proteins i.e. spectrin and band 3, which showed intensities of 10^9^–10^10^. Therefore, the majority of proteins that were significantly changed in DBA patients were of medium to high abundance and well above the limit of detection for low abundance proteins (approximately 10^4^ signal intensity).

To determine whether proteins with altered abundance levels belonged to specific pathways or processes, these proteins were further characterized using gene ontology functional annotation with DAVID (http://david.abcc.ncifcrf.gov/home) (Table S1 in File S1). Proteins that were significantly increased in DBA patients involved processes related to antigen presentation via the major histocompatibility class I pathway, cell cycle processing, and immune response. Significantly decreased proteins were categorized into oxygen transport and response to oxidative stress.

### Immunoblot Validation

**Figure 3:**
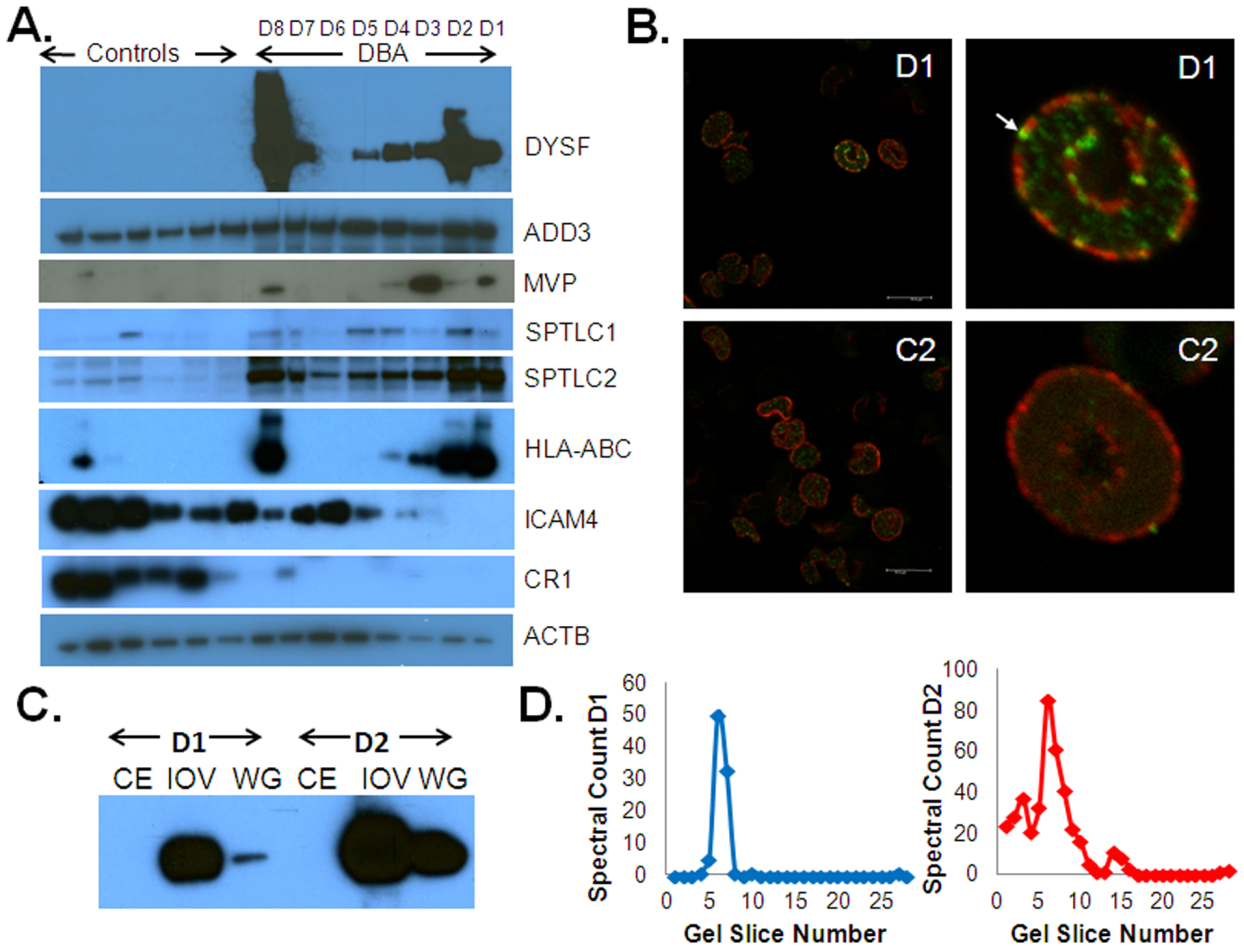
Immunoblot validation and characterization of dysferlin. A) Western blot of several proteins that were increased or decreased in DBA patients including dysferlin (DYSF), gamma-adducin (ADD3), major vault protein (MVP), serine palmitoyltransferase 1 and 2 (SPTLC1 and SPTLC2), HLA-ABC, and intercellular adhesion molecule 4 (ICAM4) with β-actin (ACTB) used as a loading control. Six individual control samples that were included in the pooled controls for proteomic analysis were used for comparison to individual DBA patient samples. B) Confocal images of intact RBCs from DBA patient D1 and healthy control C2 showing localization of dysferlin (green) at the membrane where spectrin (red) is localized. Dysferlin fluorescence signal in the membrane of DBA RBCs is present in punctuate patterns and is evident above the autofluorescent background seen in the cytoplasm as highlighted in the zoomed images on the right panel. C) Western blot showing the enrichment of dysferlin in the RBC ghosts (WG) and the inside-out vesicles (IOV) but not in the RBC membrane cytoskeletal extract (CE) of DBA patients D1 and D2. D) Distribution of dysferlin spectral counts versus gel slice number for DBA samples D1 and D2. Higher abundance of dysferlin in D1 with high molecular weight dysferlin crosslinks and low mass protein fragments correlate with immunoblots shown in panel A.

Immunoblot validation was performed for proteins of interest where suitable antibodies were available. RBC ghost preparations from 18 healthy controls and nine transfusion-independent DBA patients (Table 1) were analyzed. Figure 3A is a representative immunoblot analysis of six controls and eight DBA patients, four of whom were used in the LC-MS/MS analysis. Dysferlin was present, although at variable levels, in all white ghost preparations from DBA patients but was not detectable in any of the 18 healthy control RBC membranes tested. Dysferlin was barely detectable in DBA patient D6 with the conditions shown in Figure 3A, however, subsequent analysis using more extensive reaction conditions showed a clear band, indicating dysferlin was present albeit at a much lower level than in RBC membranes from other patients (data not shown). ADD3 was also consistently increased in all DBA samples tested compared to normal controls, although to a lesser extent, which is consistent with the relatively low fold changes in the proteomic data (ranging from 2.3–6.4 fold). MVP was largely absent in control white ghost samples, but variable protein levels were found in five of the DBA patient samples (Figure 3A). The levels of SPTLC1 were increased in DBA patients, with the majority of controls showing little to no SPTLC1. A distinct increase in SPTLC2 levels can be seen in all of the DBA patients tested compared to the low levels seen in the controls. Additionally, HLA-ABC was found to be present in variable amounts in the white ghost fractions of six DBA patients as well as two control samples. ICAM4 can be seen with variable abundance in the control samples but is present at low to moderate levels in six DBA patients and cannot be detected in two of the DBA patients. CR1 was largely absent in DBA patients whereas moderate levels were detectable in all of the controls, which is consistent with the proteomic data.

### Dysferlin in the RBC Membrane

The most striking result was the identification of dysferlin in the RBC membrane of DBA patients since it has never been described to be present in the RBC membrane. Dysferlin has been well studied because of its involvement in forms of muscular dystrophy but it has never been reported to be present in the RBC membrane in previous proteome analyses. [24], [25], [26], [27] Consistent with these earlier results, we did not detect dysferlin in any of the control donor RBC membranes. We used confocal microscopy to evaluate the location of dysferlin in the RBCs of DBA patients. Figure 3B shows representative images of dysferlin in RBC membranes of DBA patient D1, as seen with immunostaining of dysferlin (green) relative to spectrin as a control (red). Consistent with the proteome analyses, confocal microscopy of RBC from control C2 showed no detectable dysferlin. Interestingly, the levels of dysferlin in the RBCs from individual DBA patients were variable, with some cells displaying intense fluorescence, while other cells showed minimal or undetectable staining. To further evaluate dysferlin location in RBC membranes of DBA patients, we extracted spectrin and actin from ghosts and analyzed this extract and inside-out vesicles using dysferlin immunoblots (Fig. 3C). Dysferlin was present in the inside-out vesicles but was not detectable in the cytoskeletal extract which is consistent with the fact that dysferlin is a transmembrane protein.

In addition to increased depth of proteome analysis, separation of proteins by 1D gel electrophoresis prior to LC-MS/MS provided interesting information on the size distribution of proteins by comparison of spectral counts across gel slices for individual proteins. Figure 3D shows the relative abundance of dysferlin at different molecular weights for patients D1 and D2. In both cases, the majority of the protein was detected at its expected molecular weight, in the fractions corresponding to 250–200 kDa. However, for patient D2, some dysferlin was detected at lower and higher molecular weights (slices 14–16 and 1–3, respectively), which was indicative of proteolysis and covalent crosslinking of the molecule, possibly to itself, as it may be present as a homodimer. [28] The results from the immunoblot correlated with the relative abundance and size distribution of dysferlin in the LC-MS/MS data (Fig. 3).

To further investigate whether dysferlin on the RBC is a general marker of stress hematopoiesis we investigated other blood disorders for the presence of dysferlin on the RBC membrane with western blot analysis. We screened 19 patients with macrocytosis associated with various diagnoses including paroxysmal nocturnal hemoglobinuria, dyskeratosis congenita, acquired aplastic anemia, congenital neutropenia, Shwachman Diamond syndrome, diserythropoietic anemia (Gata1 mutation), and chemotherapy-related macrocytosis (Table 4). Interestingly, of all the samples tested, dysferlin was not in any other inherited bone marrow failure syndrome tested, however it was found on the RBC of four of the eight acquired aplastic anemia patients. Western blot analysis of RBC membrane preparations show low to moderate levels of dysferlin in these acquired aplastic anemia patients compared to DBA patients (Figure S3 in File S1).

**Table 4:** Cohort of Additional Bone Marrow Failure Patients Screened for Dysferlin.

Diagnosis	Age	Sex	Ethnicity	HB (g/dL)	RBC (MIL/µL)	MCV (fL)	Dysferlin*
**Acquired aplastic anemia**	**26**	**F**	**Caucasian**	**9**	**2.21**	**113**	**Y**
**Acquired aplastic anemia**	**18**	**M**	**African American**	**8.8**	**2.88**	**96.3**	**Y**
**Acquired aplastic anemia**	**47**	**M**	**Caucasian**	**11.4**	**3.17**	**100**	**Y**
**Acquired aplastic anemia**	**11**	**M**	**Caucasian**	**9.3**	**2.82**	**94.9**	**Y**
Acquired aplastic anemia	18	M	Caucasian	15.7	5.23	87.5	N
Acquired aplastic anemia	10	F	African American	11.4	3.77	91.7	N
Acquired aplastic anemia	15	F	Caucasian	–	–	–	N
Acquired aplastic anemia	19	M	Caucasian	15.1	4.51	100.6	N
Chemo-related macrocytosis	22	F	Caucasian	13.2	3.73	103	N
Congenital neutropenia	30	F	Caucasian	14.1	4.66	88	N
Diserythropoietic anemia, Gata1 mutation	20	M	African American	9.1	3.02	99.7	N
Dyskeratosis congenita	12	M	Caucasian	11.9	3.57	105.4	N
Dyskeratosis congenita	7	F	Caucasian	13	4.42	90	N
Dyskeratosis congenita	15	M	Caucasian	15.4	4.86	93.5	N
Macrocytic anemia, unknown pathogenesis	22	F	Caucasian	10.1	2.68	109	N
Paroxysmal nocturnal hemoglobinuria	18	F	African American	9.8	3.1	96.9	N
Paroxysmal nocturnal hemoglobinuria	27	F	African American	10	2.81	104	N
Paroxysmal nocturnal hemoglobinuria	53	F	Caucasian	9.5	2.75	100	N
Shwachman-diamond syndrome	4	F	Caucasian	11.6	3.65	93.6	N

Bold entries indicate detection of dysferlin. Normal ranges for Hgb: 13.5–17.5 males, 12.0–15.5 females; RBC 4.3–5.7 males, 3.9–5.0 females; MCV 80–100.

## Discussion

RBCs in DBA are distinguished from normal RBC by macrocytosis, increased fetal hemoglobin and elevated adenosine deaminase activity. Here we used 1D SDS gel separation followed by LC-MS/MS of RBC ghosts to investigate changes in relative protein levels of over one thousand RBC membrane proteins to identify a unique protein signature that further distinguishes DBA patients from normal controls. To confirm that protein changes were not due to proteins from contaminating cell types, we searched our datasets for proteins that were recently listed in a review of 50 blood cell-type specific proteins. Of the 41 proteins specific to monocytes, T cells, platelets, and neutrophils, only 6 were identified in our proteomic datasets (plasma membrane calcium transporting ATPase and tyrosine-protein kinase *SYK* from monocytes, JAM-A, Ras-related protein Rab-6B, and Syntaxin-11 from platelets, and cathelicidin antimicrobial peptide precursor from neutrophils.) Additionally, we did not detect the transferrin receptor, a known reticulocyte marker. Furthermore, contaminant proteins were not found to be significantly changed, indicating they were uniformly distributed across different sample preparations.

The DBA patients analyzed in this study harbor different ribosomal protein mutations, with patients D3 and D4 on prednisone treatment. Patients D1 and D2 were without treatment at the time of this study, however they still present with macrocytic anemia (Table 1) and have received transfusions in the past. Comparison of results with the ribosomal mutations and patient hematological status did not reveal any obvious trends in protein abundance as a function of treatment or mutation. Only one protein, TAP2, was significantly increased in patients D3 and D4 only, indicating that the majority of proteins were not induced from steroid treatment alone. Interestingly, two patients with RPL5 mutations showed very high levels of dysferlin compared to the other DBA patients. However, more patient samples with differing ribosomal protein mutations are needed for definitive analysis.

The most surprising result is the identification of dysferlin on the RBC membrane of DBA patients because it has never been reported to be present on the RBC and it does not have a well-defined role in hematopoiesis. Dysferlin is a 230 kDa protein with a type two single-pass membrane spanning domain and seven calcium-binding C2 domains. It is normally highly expressed in muscle and mutations in this protein can lead to adult-onset muscular dystrophies, including Limb Girdle Muscular Dystrophy type 2B, [29] Myoshi Myopathy, [30] and Distal Anterior Compartment Myopathy. [31] Dysferlin is also found at lower expression levels in monocytes, endothelium, brain, kidney, and pancreas [32].

The presence of dysferlin in the RBC membrane appears to be relatively specific to DBA. Immunostaining for dysferlin in RBC ghosts showed variable levels of dysferlin present in the RBCs of all transfusion-independent DBA patients tested but it was not found on RBCs of transfusion dependent DBA patients (data not shown), indicating that dysferlin is an integral RBC membrane protein and does not derive from white blood cell contamination or from transfer to circulating RBCs by monocytes. Based on the accepted copy numbers of α-spectrin and band 3 per red cell (2.42×10^5^ and 1.2×10^6^, respectively) [33] we can estimate the copy numbers of dysferlin in the DBA patients using the protein abundance values shown in Figure 2. These calculations yield approximate copy numbers of 1000, 4000, 2, and 100 for dysferlin in patients D1–D4, respectively. Screening other macrocytic blood disorders revealed the presence of dysferlin in the RBC of four of eight acquired aplastic anemia patients but not in any of the other 11 patients with various bone marrow failure disorders. Interestingly, one of these aplastic anemia patients has slightly higher eADA activity and a copy number neutral 5q loss of heterozygosity in the region of the *RPS14* gene, raising the possibility of a functional connection between RP haploinsufficiency and dysferlin accumulation in RBCs.

At this point, the potential roles of dysferlin on RBCs or in RBC maturation are mainly speculative, whether these are specific to the DBA pathology and impaired ribosome biogenesis, or whether the expression of dysferlin allows DBA erythropoiesis to produce circulating RBCs and rescue RBC production in the context of impaired ribosome biogenesis remains to be determined. The majority of studies on dysferlin function have been in the context of muscular dystrophy. In muscle, dysferlin was found to accumulate at the site of injury and is involved in membrane repair. [34] The normal function of dysferlin in monocytes is not well characterized, however, dysferlin deficient monocytes displayed increased phagocytosis. [35] A recent report showed that dysferlin interacts with components of the focal adhesion complex, implicating a role in cellular adhesion [36].

Several MHC class I proteins and MVP were present on the RBC membranes of DBA patients but not of healthy controls. MHC class I proteins are found on nucleated cells and are therefore not normally present on the surface of mature RBCs. However, several studies have found MHC class I molecules on the RBC of patients with systemic lupus erythematosus, [37], [38] mononucleosis, [39] HIV, and interferon-α treatment. [40] Furthermore, a decrease in CR1 is also associated with systemic lupus erythematosus, further suggesting a link between DBA and an increased immune response. [41] Interestingly, a link between p53 and MVP has been described in which MVP was upregulated during p53 over-expression in human diploid fibroblasts and p53 was shown to directly bind to the MVP promoter. [42] Therefore, the increased activation of p53 in DBA erythroid progenitors [7] may explain the increase of MVP in DBA RBCs.

The results presented herein demonstrate that comparative proteome analysis of RBC membranes sensitively compares the abundance of a thousand RBC proteins and this unbiased approach revealed an unexpected expression of red cell membrane proteins on DBA RBC that would not have been discovered otherwise. The mechanism leading to the differences in the RBC proteome in DBA and the function of these proteins in the differentiation and maturation of DBA RBCs will be the subject of further investigation, which may be relevant to understanding DBA pathogenesis, diagnosis and treatment.

## Supporting Information

File S1 **Combined file of supporting figures and tables.** Figure S1: Derivation of 95% confidence intervals for two sets of donor pools using normalized protein intensities. A) Plot of log10 standard deviation and mean protein intensities with exponential decay fit for donor pools C1 and C2 for comparison of patients D1 and D2, r^2^ = 0.8665. B) Plot of log10 standard deviation and mean protein intensities with exponential decay fit for donor pools C3 and C4 for comparison of patients D3 and D4, r^2^ = 0.8810. Figure S2: Normalized log10 intensity plot of DBA patients D1–4 versus appropriate controls pools with a minimum detectable signal threshold of 1×10^4^. Figure S3: Western blot analysis of RBC membrane preparations showing dysferlin and actin as a loading control for dysferlin-positive acquired aplastic anemia patients (labeled using their ages from Table 4 in the manuscript) for comparison to DBA patients D1–D4. Table S1: Functional Annotation Classification of Significantly Changed Proteins.
(DOCX)
