# Supplementary material for: Dysferlin and Other Non-Red Cell Proteins Accumulate in the Red Cell Membrane of Diamond-Blackfan Anemia Patients
Source: PLoS One. 2014 Jan 14;9(1):e85504. doi: 10.1371/journal.pone.0085504 (PMC3891812; doi:10.1371/journal.pone.0085504)
Supplement: File S1 — Combined file of supporting figures and tables. Figure S1: Derivation of 95% confidence intervals for two sets of donor pools using normalized protein intensities. A) Plot of log10 standard deviation and mean protein intensities with exponential decay fit for donor pools C1 and C2 for comparison of patients D1 and D2, r2 = 0.8665. B) Plot of log10 standard deviation and mean protein intensities with exponential decay fit for donor pools C3 and C4 for comparison of patients D3 and D4, r2 = 0.8810. Figure S2: Normalized log10 intensity plot of DBA patients D1–4 versus appropriate controls pools with a minimum detectable signal threshold of 1×10^4^. Figure S3: Western blot analysis of RBC membrane preparations showing dysferlin and actin as a loading control for dysferlin-positive acquired aplastic anemia patients (labeled using their ages from Table 4 in the manuscript) for comparison to DBA patients D1–D4. Table S1: Functional Annotation Classification of Significantly Changed Proteins. (DOCX) [file pone.0085504.s001.docx]

**Dysferlin and other non-red cell proteins accumulate in the red cell membrane of Diamond-Blackfan Anemia patients**

Supporting Information

**Figure S1.**

**
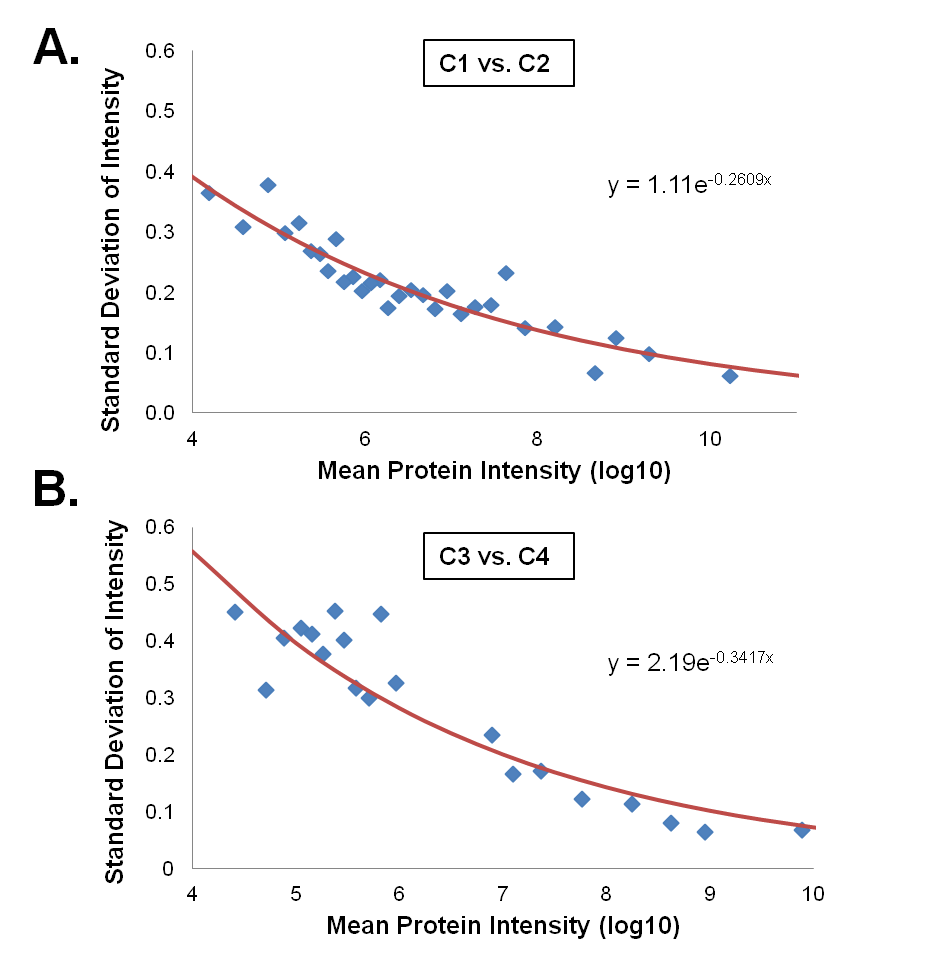
**

**Figure S2.**


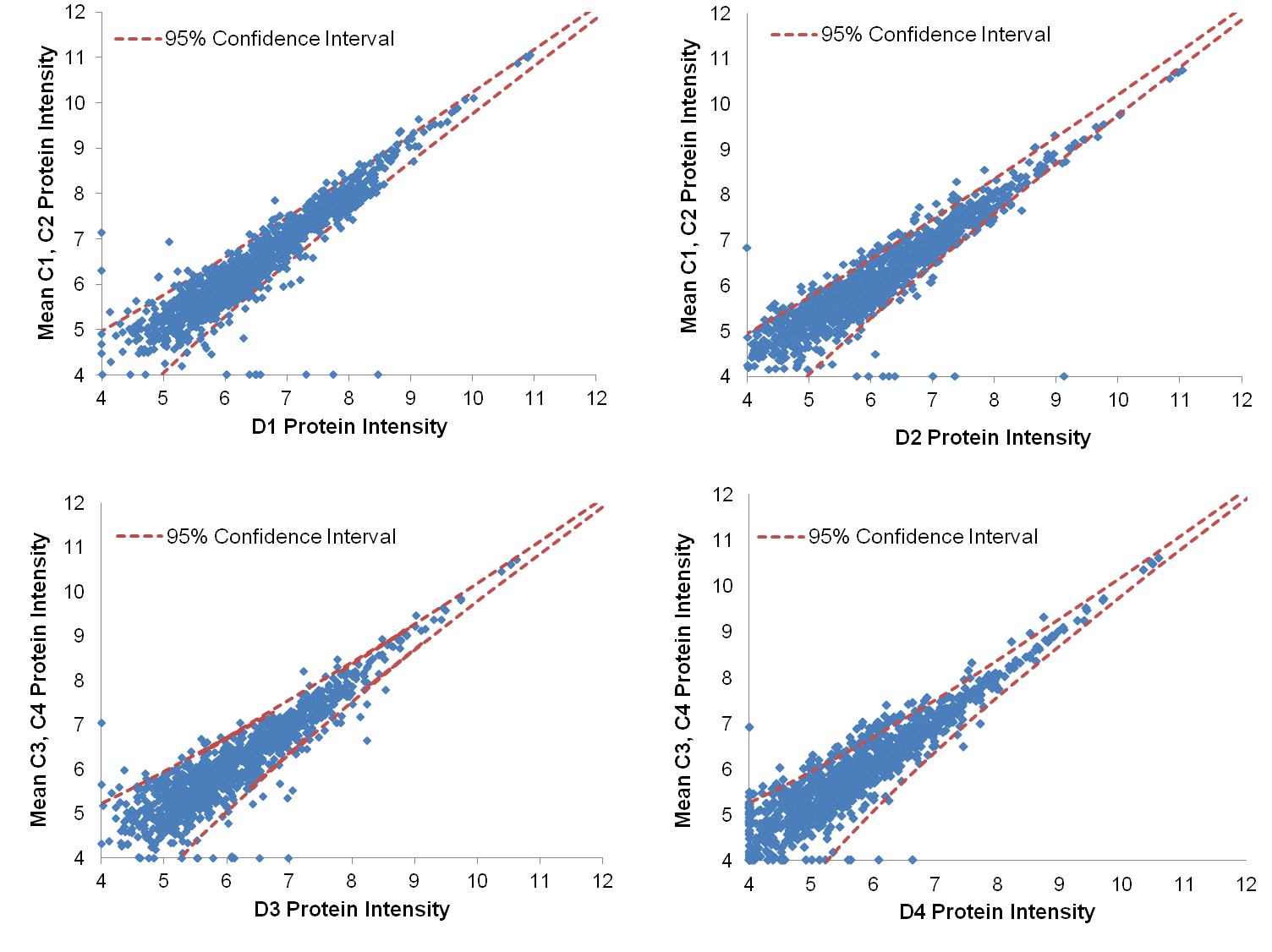


**Figure S3.**


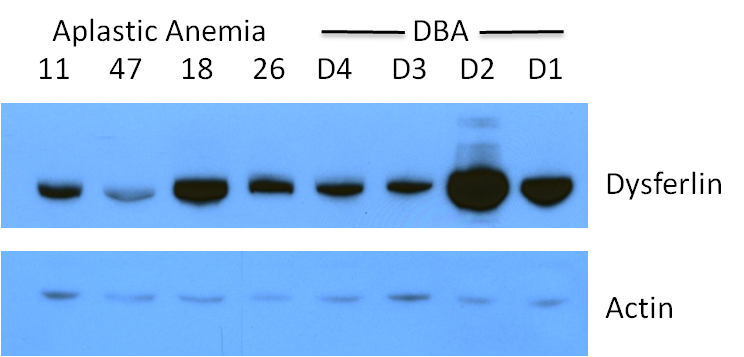


**Table S1.** Functional Annotation Classification of Significantly Changed Proteins

| **Increased in DBA** |  |  |  |  |
| --- | --- | --- | --- | --- |
| **GO Term** | **Count** | **% of Total** | **p-value** | **Genes** |
| Antigen processing and presentation | 7 | 20 | 2.89E-08 | TAP2, HLA-A, HLA-B, PSMB8, PSMB9, TAPBP, B2M |
| Antigen processing and presentation of peptide antigen via MHC class I | 5 | 14 | 5.26E-08 | TAP2, HLA-A, HLA-B, TAPBP, B2M |
| Cell cycle process | 8 | 23 | 2.34E-04 | SBDS, DCTN3, THBS1, DCTN1, PSMD8, PSMB8, MYH10, PSMB9 |
| Antigen processing and presentation of exogenous peptide antigen | 3 | 9 | 2.76E-04 | TAP2, TAPBP, B2M |
| Immune response | 8 | 2 | 7.82E-04 | TAP2, HLA-A, HLA-B, THBS1, PSMB8, PSMB9, TAPBP, B2M |
| **Decreased in DBA** |  |  |  |  |
| Oxygen transport | 3 | 8 | 3.65E-04 | HBA1, HBB, HBD |
| Response to oxidative stress | 5 | 13 | 4.46E-04 | PRDX2, CA3, CAT, PRDX1, GCLM |
| Hydrogen peroxide catabolic process | 3 | 8 | 6.33E-04 | PRDX2, CAT, PRDX1 |
